# Small scale migration along the interoceanic highway in Madre de Dios, Peru: an exploration of community perceptions and dynamics due to migration

**DOI:** 10.1186/s12914-018-0152-8

**Published:** 2018-02-12

**Authors:** Kelly E Jensen, Nehal N. Naik, Christina O’Neal, Gabriela Salmón-Mulanovich, Amy R. Riley-Powell, Gwenyth O. Lee, Stella M. Hartinger, Daniel G. Bausch, Valerie A. Paz-Soldan

**Affiliations:** 1 Tulane University School of Medicine, 1430 Tulane Ave, New Orleans, LA 70112 USA; 2 Tulane University School of Public Health and Tropical Medicine, 1440 Canal St, New Orleans, LA 70112 USA; 3 School of Medicine, Virginia Commonwealth University, 1201 E Marshall St, Richmond, VA 23298 USA; 4 Naval Medical Research Unit No. 6, Callao, Peru; 5 Universidad Peruana Cayetano Heredia, Ave Honorio Delgado 430, Distrito de Lima, Peru; 6 School of Public Health, University of Michigan, 1415 Washington Heights, Ann Arbor, MI 48109 USA; 7 Swiss Tropical and Public Health Institute, Socinstrasse 57, 4051 Basel, Switzerland; 8 Bellevue, USA

**Keywords:** Rural migration, Economic growth and development, Community perceptions

## Abstract

**Background:**

Madre de Dios, a southern state in the Peruvian Amazon basin, has experienced rapid development as well as an influx of migrants since the construction of the Interoceanic Highway (IOH) connecting Brazil, Bolivia, and the Peruvian coast. We explored perceptions of migration and development in up to eight communities along the IOH in Madre de Dios following construction of the highway.

**Methods:**

We conducted a multiple methods study involving focus group (FG) discussions and interviews with key informants (KIs) in eight communities in Madre de Dios. The data was used to develop and apply a survey on demographics, financial, personal, social, human, and physical capital in four communities between February 2014 and March 2015.

**Results:**

We conducted 12 FGs and 34 KI interviews. A total of 522 people participated in the survey. Comparing migrants (those who had moved to the area after construction of the IOH) and non-migrants, we found no difference in food security or access to health services. The majority (67.6%) of respondents from both groups reported that illness was their primary threat to well-being. Non-migrants owned more land than migrants (*p* < 0.001), were more likely to have piped water directly in their home (*p* = 0.046), and were more likely to participate in community groups (*p* = 0.012). Looking at perceptions about migrants, KIs and FGs discussed both positive perceptions of migrants (increased cultural exchange and new technology) and negative perceptions (increased drugs and alcohol in their communities and a lack of investment in the community). Both migrants and non-migrants reported trusting the local government more than the national government.

**Conclusions:**

Although we hypothesized that migrants would have decreased access to food, water, health services, and land relative to non-migrants, our results show that the only significant differences were in land ownership and water access. Efforts to improve community infrastructure should be carried out at the local level and focus on improving issues reported by both groups, such as potable water, sewage, and increased access to health services. Furthermore, an emphasis on community cohesion, ensuring land rights, and increasing long-term job opportunities should help ease tensions between migrants and non-migrants.

**Electronic supplementary material:**

The online version of this article (10.1186/s12914-018-0152-8) contains supplementary material, which is available to authorized users.

## Background

**Fig. 1:**
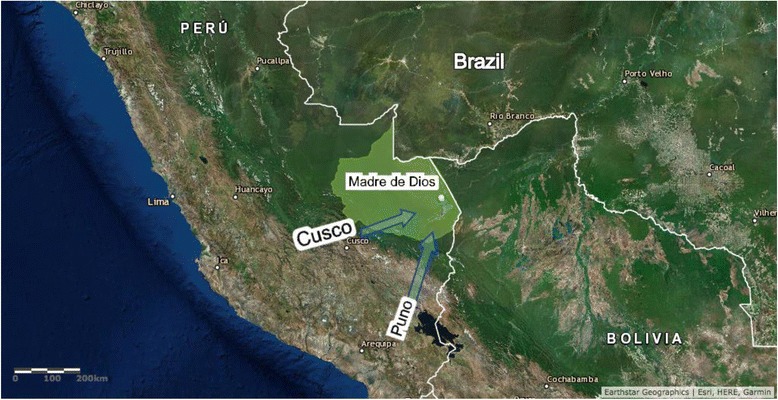
Map illustrating the major migration patterns to Madre de Dios following the construction of the Interoceanic Highway. Created Using: ArcGIS Online [Internet]. Redlands, CA: Environmental Systems Research Institute (ESRI); Available from: www.esri.com

Madre de Dios, a southern state in the Peruvian Amazon river basin, has been a destination of internal migration since the 1960s, following the construction of the first road into the province [1]. Migration into this area has since increased steadily due to the availability of natural resources such as rubber, Brazil nuts, animal skins, fine wood, and gold. In the 2000s, the province saw a sudden influx of new migrants as a result of the construction of the Interoceanic Highway (IOH) [1, 2] and gold mining, much or all of it illegal [3]. The construction of the IOH, which connects the Atlantic ports of Brazil to the Pacific ports of Peru, was part of the Initiative for Integration of Regional Infrastructure in South America to increase cross-border infrastructure and economic development [1, 4]. Construction began in Brazil in 2002, in Peru in 2006, and was completed in 2011 [2, 5]. Unlike other internal migration movements in Peru, which have generally been rural to urban [6], migration in Madre de Dios has been rural to rural. Migrants are mainly arriving from other parts of the Peruvian Amazon, as well as from the Andean regions of Cusco and Puno which have possible Quechua or Aymara culture and language influences (Fig. 1). It is important to note that Peru is characterized by three distinct geographic regions: the coast, the Andes (mountains), and the rainforest. Although Spanish is considered the national language, each region has distinct characteristics leading to different lifestyles, as well as different indigenous and local groups.

Previous migration studies have focused on the economic rational for migration from rural to urban areas [7] or the capacity of cities to accommodate new migrants [8]. The term “migrant” has no internationally recognized definition [9]. Varying classifications in the literature have included non-native birthplace, citizenship or legal status, or reason for migration [9, 10]. In Latin America, urbanization is driving economic migration [6]. The construction of the IOH, leading to increased economic opportunities (such as logging or gold mining), is the main driving factor for migration to Madre de Dios. Therefore, in this study we define a migrant as a community member who self-identified as living in the community after the construction of the IOH began in Peru (equivalent to having moved to the community in or after 2006).

Migrants face many challenges upon relocation, including access to government programs, especially for health care and other public services. Migrants also tend to earn disproportionately lower incomes and shoulder higher economic costs [11, 12]. Preliminary qualitative data collected by our working group suggested that there was conflict between migrants and non-migrants within the communities in Madre de Dios, leading to a distinction between the two groups, and to our decision to examine this issue further. In Peru, studies such as the PERU MIGRANT project have started to look at health disparities between rural to urban migrants, but little has been done to look at the community as a whole, and few studies examine rural to rural migration, particularly in remote areas of the rainforest, and how this affects the lives of the migrants and the long-term residents [9, 10].

The study “Effects of Anthropogenic Habitat Perturbation on Rodent Population Dynamics and Risk of Rodent-Borne Diseases” is a five-year multisite investigation designed to examine the impact of anthropogenic land use on habitat integrity, biodiversity loss, and the risk of rodent-borne diseases [2]. Investigation of community perceptions of changes related to the IOH were included as part of the study. Here we report on community perceptions of migration in the Madre de Dios region after construction of the IOH and the issues communities face following rapid development. Findings from this study can be used to inform policy and programmatic interventions to improve local services, infrastructure, and the wellbeing of residents in these rapidly changing communities.

## Methods

### Sampling

Purposive sampling was used to select communities based on community size, proximity to the IOH, accessibility to Puerto Maldonado, and proximity to communities included in a prior pilot study [13]. Community sizes ranged from 250 to 2500 residents based on 2007 census data [14].

### Study design

**Fig. 2:**
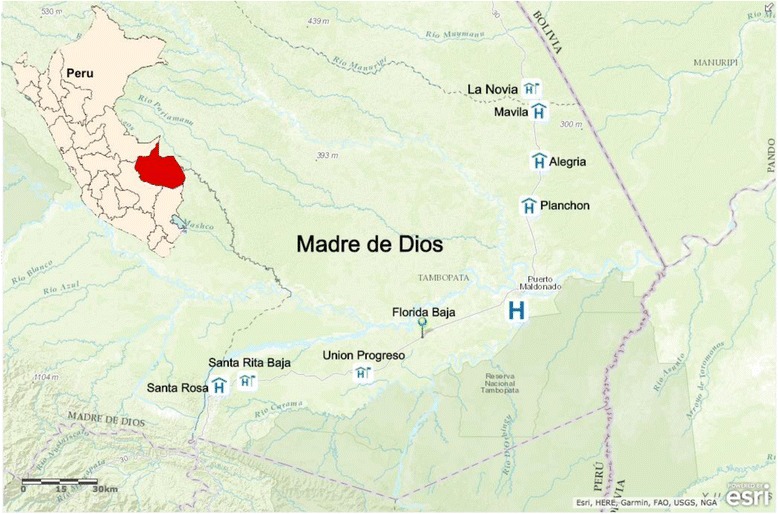
Map of Madre de Dios and the surrounding eight communities involved in the study. Created Using: ArcGIS Online [Internet]. Redlands, CA: Environmental Systems Research Institute (ESRI); Available from: www.esri.com

Data collection occurred in two phases: The first phase took place in 2014 and consisted of qualitative methods to explore community member’s perspectives of their health and wellbeing. We conducted focus group (FG) discussions and key informant (KI) interviews in eight communities located along the IOH in Madre de Dios, Peru. Half of the participating communities were northwest of Puerto Maldonado (the Region’s capital city) and the other half were southwest of the capital (Fig. 2). The second phase took place in 2015 and comprised the application of a survey, which was developed using data from 2014, and additional KI interviews in four of these communities (two northwest and two southwest of the capital). All qualitative methods continued until saturation – the point at which no new themes are uncovered – was reached [15].

### Focus group discussions

In phase one we used purposive sampling to identify individuals for FG participation. Due to the nature of our data collection (field workers going door to door during the day), our sample was primarily made up of women since, in this region, men tended to be out of the house working during the day. These women were often in charge of managing their household’s health and had often been living in the area for over five years. We targeted community members who were able to comment on changes since the construction of the IOH and influx of new residents. This group included individuals living in the area both before, and around the time, the IOH was constructed. FG participants were recruited one-to-two days prior to scheduled FG dates through community authorities and the research team. However, due to low attendance rates, additional available individuals in the community were recruited the same day the FG took place. Each FG was facilitated by a trained Peruvian anthropologist with a FG guide used to direct the discussions and included questions about health, well-being, rodents, the IOH, education, safety, and food security. Detailed notes were compiled at the end of each day and FG data were summarized manually and then thematically analyzed with Dedoose software [16] using a grounded theory approach.

### Key informant interviews

All KIs were identified by local collaborators using purposive and snowball sampling: we started by identifying community authorities and health personnel who then suggested other individuals in the community who act in leadership roles. We developed an interview guide that included questions about the community and changes observed within the communities over the past 5–10 years (Additional file 1). The FG facilitator led the KI interviews in 2014 and another trained researcher conducted the KI interviews in 2015 with an accompanying note taker. We made detailed notes for all interviews. We summarized KI interview data manually and then thematically analyzed the data in ‘Dedoose’ [16]. Themes are described within the text and quotes used where relevant.

### Survey

The survey, conducted in 2015 was comprised of six sections relating to different domains mapping the five forms of capital (financial, personal, social, human, and physical) emphasized in the livelihood approach [17]. This was consistent with themes that were identified in the 2014 qualitative data. We administered the survey door-to-door by trained fieldworkers under the supervision of an experienced field coordinator. Due to controversy about gold mining in Madre de Dios and possible legal repercussions, local authorities and KIs requested that no information about gold mining be elicited during the survey.

### Data analysis

We entered and analyzed survey data using STATA 14.1 [18]. We performed bivariate analysis based on migrant versus non-migrant status using Pearson’s chi square test or Fisher’s exact test, as required, for categorical variables and independent sample t-test comparing means for continuous variables. We used regression analysis to examine the associations between variables of interest, while controlling for age, sex, and wealth index. We set statistical significance at a two-tailed *p*-value < 0.05.

### Variables

We developed a wealth index using diverse indicators of financial capital, including housing quality, durable consumer goods, and housing services/utilities as a decimal score between 0 and 100 [19].

### Ethics

The study was approved by the Institutional Review Boards of the US Naval Medical Research Unit No. 6 (NAMRU-6) (NAMRU6.2013.0025), the Universidad Peruana Cayetano Heredia (62162), and Tulane School of Public Health and Tropical Medicine (545719). In addition, we contacted local authorities and community leaders from each study area, who also agreed to provide support. We obtained written informed consent from all participants.

## Results

### Focus groups

We conducted 12 FGs, with participation from a total of 83 community members from eight communities. The majority of FGs were with female participants (68% of FG participants were female) due to our experience that: 1) women often participate more when men are not present, and 2) in this region, women (primarily mothers) tend to be the ones who manage household illnesses, which were the focus of the study. Due to our interest in exploring life before and after the IOH, ten of the FGs were composed exclusively of long-term residents to explore community changes and historical perceptions. All FG participants were over 18 years old.

### Key informant interviews

We conducted 34 KI interviews with community leaders and healthcare personnel, 21 in February 2014 (between one and four KI interviews in each of the eight communities) and 13 in March 2015 (three to four KI interviews in each of the four communities). No recordings were available from interviews in 2014 and four of the interviewees in 2015 did not consent to be recorded. For KIs who did not consent to recordings (though all consented to study participation), summary notes were used to identify general themes. Transcripts were used from nine KI interviews from March 2015: Santa Rosa, La Novia, Alegría, and Florida Baja (*N* = 2, 2, 3, and 2, respectively). We categorized occupations of KIs into four main groups: elected officials (*N* = 5), health care professionals (*N* = 2), education professionals (*N* = 1), and legal professionals (*N* = 1).

### Survey

**Fig. 3:**
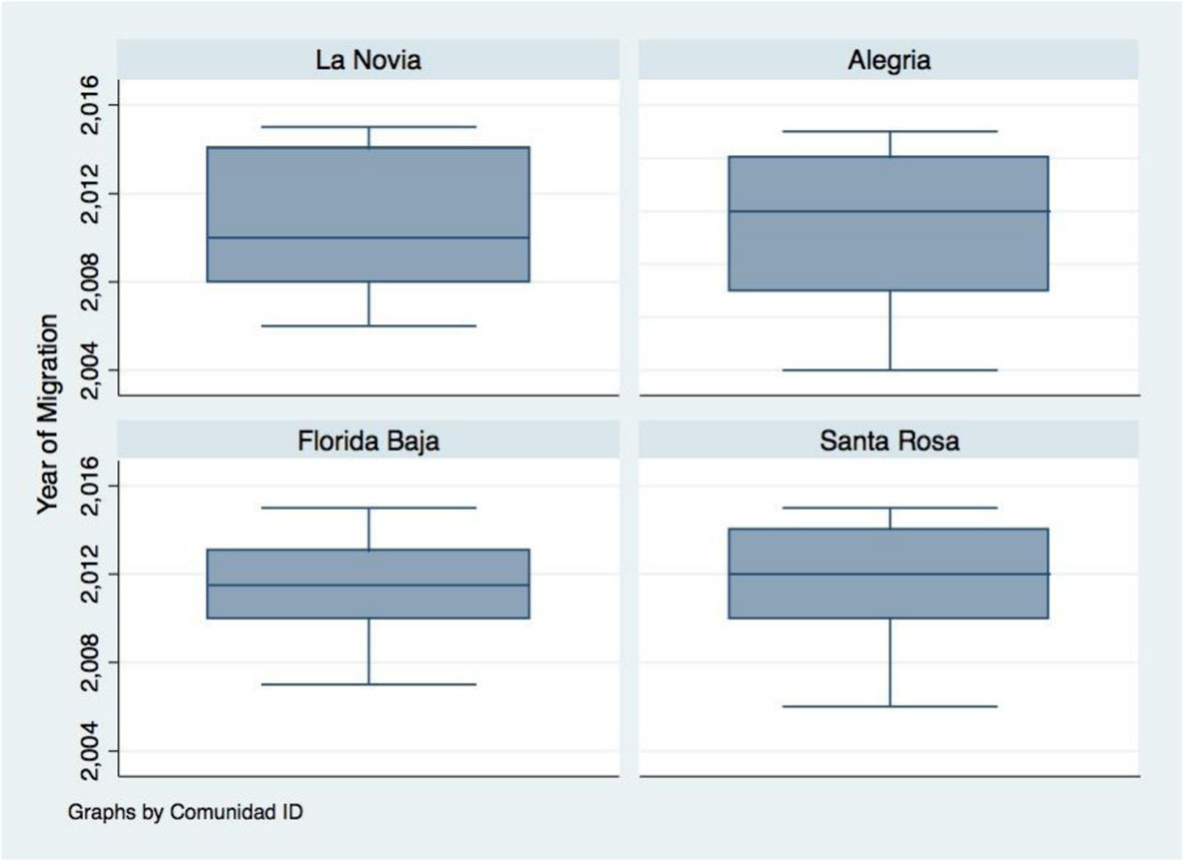
Comparison of arrival date by community for migrants arriving between 2000 and 2015

A high proportion of houses (41%) were found to be uninhabited when attempting to apply the survey, 6% declined participation, and 8% could not talk to the surveyors at the time of data collection. There were a total of 522 survey respondents, answering on behalf of themselves and the individuals in their homes. Only individual respondent answers were used in this analysis unless otherwise indicated (head of households were asked about threats to health and wellbeing that affected both themselves and their family members). A total of 202 (38.7%) of respondents were defined as non-migrants and 320 (61.3%) as migrants (Table 1). The average arrival date of migrants in La Novia was in 2010, while in other communities it was around 2012 (Fig. 3). We found significant differences between migrants and non-migrants with regard to age and sex, but not civil status, religion, language, or level of education. Common occupational activities included forestry, agriculture/farming, and commerce. There was no significant difference in occupation type between migrants and non-migrants.

**Table 1:** Demographics

	Sample *N* = 522	Non-Migrant *N* = 202	Migrant *N* = 320	*P*-value
Age, mean (SD)	36.0 (0.6)	40.6 (1.0)	33.0 (0.6)	< 0.001
Female, no. (%)	371 (71.1%)	133 (65.8%)	238 (74.4%)	0.036
Civil Status				0.088
Cohabitating/married	406 (77.8%)	151 (74.7%)	255 (79.7%)	
Separated/widowed/divorced	60 (11.5%)	31 (15.3%)	29 (9.1%)	
Single	56 (10.7%)	20 (9.9%)	36 (11.3%)	
	Sample *N* = 510	Non-Migrant *N* = 196	Migrant *N* = 314	*P*-value
Occupation				0.203
Forestry	157 (30.8%)	63 (32.1%)	94 (29.9%)	
Agriculture/farming	148 (29.0%)	47 (24.0%)	101 (32.1%)	
Commerce	45 (8.8%)	18 (9.2%)	27 (8.6%)	
Transport	40 (7.8%)	15 (7.7%)	25 (8.0%)	
Construction/mechanic	39 (7.7%)	13 (6.6%)	26 (8.3%)	
Household activities	23 (4.5%)	13 (6.6%)	10 (3.2%)	
Management/professional	15 (2.9%)	5 (2.5%)	10 (3.2%)	
Other	43 (8.4%)	22 (11.2%)	21 (6.7%)

### Examining five livelihood capitals, stratified by migrant vs non-migrant

During exploratory analysis in 2014, the clear—and at times tense—dynamic between more recent and long-term residents was apparent, prompting this analysis. We explored community perceptions regarding recent migrants more carefully in FG discussions, KI interviews, and surveys conducted in 2015. We used the livelihood capital framework [13] to design questions, as well as to present our findings, stratifying our findings at each level of the livelihood capitals by migrant status to examine any differences. See Tables 2, 3, 4, 5, 6, 7, 8, 9 and 10 for a summary of the findings by subheading within each type of capital.

**Table 2:** Physical capital: Food access

	Sample *N* = 522	Non-Migrant *N* = 202	Migrant *N* = 320	*P*-value
Describe the food situation at home in the last 12 months:				0.239
We always eat sufficiently	153 (29.3%)	60 (29.7%)	93 (29.1%)	
Eat sufficiently, but not always	300 (57.5%)	123 (60.9%)	177 (55.3%)	
Sometimes we do not eat sufficiently	58 (11.1%)	16 (7.9%)	42 (13.1%)	
Frequently we do not eat sufficiently	11 (2.1%)	3 (1.5%)	8 (2.5%)	
	Sample *N* = 69	Non-Migrant *N* = 19	Migrant *N* = 50	*P*-value
Reasons for insufficient food:				0.699
I do not have enough money for food	60 (87.0%)	16 (84.2%)	44 (88.0%)	
Other	9 (13.0%)	3 (15.8%)	6 (12.0%)	
Worried about running out of food before you can acquire more during the last 12 months:				0.351
Never	10 (14.5%)	4 (21.1%)	6 (12.0%)	
Total Yes	59 (85.5%)	15 (78.9%)	44 (88.0%)	
Yes - rarely/sometimes	34 (49.3%)	7 (36.8%)	27 (54.0%)	
Yes - frequently/always	25 (36.2%)	8 (42.1%)	17 (34.0%)	
Reaction to insufficient food at home:				0.487
Get a loan from a store, family or friends	37 (53.6%)	9 (47.4%)	28 (56.0%)	
Reduce rations or not eat	23 (33.3%)	6 (31.6%)	17 (34.0%)	
Other	9 (13.1%)	4 (21.0%)	5 (10.0%)

**Table 3:** Physical capital: Water access

	Sample *N* = 522	Non-Migrant *N* = 202	Migrant *N* = 320	*P*-value
Primary water supply for family:				0.046
Tubed/piped water source	439 (84.1%)	178 (88.1%)	261 (81.6%)	
Open/natural water source	83 (15.9%)	24 (11.9%)	59 (18.4%)	
Primary drinking water source for family:				0.020
Tubed/piped water source	441 (84.5%)	180 (89.1%)	261 (81.6%)	
Open/natural water source	81 (15.5%)	22 (10.9%)	59 (18.4%)	
Treat water to make it safer to drink:				0.045
Yes	360 (69.0%)	129 (63.9%)	231 (72.2%)	
Method used to treat water to make it safer to drink:				0.200
Boil the water	295 (56.5%)	108 (53.5%)	187 (58.5%)	
Chlorine/Bleach	62 (11.9%)	20 (9.9%)	42 (13.1%)	
None	162 (31.0%)	73 (36.1%)	89 (27.8%)	
Other	3 (0.6%)	1 (0.5%)	2 (0.6%)

**Table 4:** Human capital: Wellbeing

	Total *N* = 237	Non-Migrant *N* = 97	Migrant *N* = 140	*P*-value
Seek treatment or advice when you have an illness:				0.890
Yes	204 (86.1%)	78 (80.4%)	126 (90.0%)	
If YES:				0.189
Treatment site:				
Public health services	165 (80.9%)	61 (78.2%)	104 (82.5%)	
Private health services	21 (10.3%)	12 (15.4%)	9 (7.2%)	
Non-regulated health services	18 (8.8%)	5 (6.4%)	13 (10.3%)	
Pay for any treatment:				0.004
Yes	113 (55.4%)	54 (69.2%)	59 (46.8%)	
	Total *N* = 203	Non-migrant *N* = 74	Migrant *N* = 129	*P*-value
Distance from home to treatment site:				0.008
Less than 30 min	92 (45.3%)	24 (30.8%)	68 (54.4%)	
Less than 1 h	30 (14.8%)	13 (16.7%)	17 (13.6%)	
Less than 2 h	29 (14.3%)	13 (16.7%)	16 (12.8%)	
More than 2 h	52 (25.6%)	28 (35.9%)	27 (19.2%)

**Table 5:** Personal capital: Threats to health and wellbeing

	Total (*n* = 1265)*	Non-migrant (*n* = 493)	Migrant (*n* = 772)	*P*-Value
Principal threat to the health and wellbeing of the family				0.040
Illness	855 (67.6%)	322 (65.3%)	533 (69.0%)	
Injuries/Accidents	199 (15.7%)	74 (15.0%)	125 (16.2%)	
Poor Hygiene/Nutrition	96 (7.8%)	40 (8.1%)	58 (7.5%)	
Drugs/Alcohol	49 (3.9%)	29 (5.9%)	20 (2.6%)	
Other	64 (5.0%)	28 (5.7%)	36 (4.6%)	

*Head of household answered for themselves and their family members

**Table 6:** Financial capital: Land ownership

	Total (*N* = 518)	Non-Migrant (*N* = 201)	Migrant (*N* = 317)	*P*-Value
Hectares of land owned:				< 0.001
Less than 1	355 (68.5%)	116 (57.7%)	239 (75.4%)	
1 to 10	43 (8.3%)	9 (4.5%)	34 (10.7%)	
11 to 20	42 (8.1%)	19 (9.4%)	23 (7.3%)	
More than 20	78 (15.1%)	57 (28.4%)	21 (6.6%)

**Table 7:** Personal capital: Safety and threats to security/wellbeing

	Sample (*N* = 522)	Non-Migrant (*N* = 202)	Migrant (*N* = 320)	*P*-value
Feel safe in your community:				0.211
Yes	329 (63.0%)	132 (65.3%)	197 (61.6%)	
Feel more exposed to insecurity in the last 5 years:				< 0.001
Yes	303 (58.0%)	131 (64.9%)	172 (53.8%)	
Taken additional security measures:				0.051
Yes	136 (26.1%)	62 (30.7%)	74 (23.1%)

**Table 8:** Social capital: Reasons for migration

	Sample (*N* = 461)*	Non-Migrant (*N* = 144)	Migrant (*N* = 317)	*P*-value
Reason for moving here:				< 0.001
More work opportunities/land access (self or family)	358 (77.7%)	94 (65.3%)	264 (83.3%)	
Better life conditions (location/family presence)	73 (15.8%)	32 (22.2%)	41 (12.9%)	
Other	30 (6.5%)	18 (12.5%)	12 (3.8%)	
	Sample (*N* = 465)*	Non-Migrant (*N* = 145)	Migrant (*N* = 320)	*P*-value
Moved with:				< 0.001
With partner/family	329 (70.8%)	75 (51.7%)	254 (79.4%)	
Alone	94 (20.2%)	46 (31.7%)	48 (15.0%)	
With friends	16 (3.4%)	9 (6.2%)	7 (2.2%)	
Other	26 (5.6%)	15 (10.4%)	11 (3.4%)	

*Answered if not born in region

**Table 9:** Social Capital: Reasons to stay

	Sample (*N* = 522)	Non-Migrant (*N* = 202)	Migrant (*N* = 320)	*P*-value
Motivation to stay:				< 0.001
Work	319 (61.1%)	84 (41.6%)	235 (73.5%)	
Family lives here	104 (20.0%)	69 (34.2%)	35 (10.9%)	
I have land here	56 (10.7%)	30 (14.8%)	26 (8.1%)	
Other	43 (8.2%)	19 (9.4%)	24 (7.5%)	
Think you’ll bring more people here:				0.063
Yes	90 (17.2%)	27 (13.4%)	63 (19.7%)	
Live here all year:				0.246
Yes	502 (96.2%)	197 (97.5%)	305 (95.3%)	
Length of time you think you’ll stay here:				< 0.001
1–5 years	168 (32.2%)	37 (18.3%)	131 (40.9%)	
6–20 years	74 (14.2%)	25 (12.4%)	49 (15.3%)	
Always	280 (53.6%)	140 (69.3%)	140 (43.8%)	
DNI listed as here:				< 0.001
Yes	334 (64.0%)	182 (90.1%)	152 (47.5%)

**Table 10:** Perceptions of community administration

	Sample *N* = 522	Non-Migrant *N* = 202	Migrant *N* = 320	*P*-value
Decision makers in the community:				0.012
Local/regional authorities	416 (79.7%)	161 (79.7%)	255 (79.7%)	
Everyone (voting)	62 (11.9%)	30 (14.8%)	32 (10.0%)	
Local leaders (organizations/households)	12 (2.3%)	6 (3.0%)	6 (1.9%)	
Does not know	32 (6.1%)	5 (2.5%)	27 (8.4%)	
Local leaders are corrupt:				< 0.001
Yes	219 (42.0%)	103 (51.0%)	116 (36.3%)	
Does not know	155 (29.7%)	35 (17.3%)	120 (37.5%)	
Regional leaders are corrupt:				0.012
Yes	304 (58.2%)	134 (66.3%)	170 (53.1%)	
Does not know	166 (31.8%)	52 (25.7%)	114 (35.6%)	
National leaders are corrupt:				0.223
Yes	376 (72.0%)	154 (76.2%)	222 (69.4%)	
Does not know	110 (21.1%)	37 (18.3%)	73 (22.8%)	
Your opinion matters, and to whom:				< 0.001
No	172 (32.9%)	46 (22.8%)	126 (39.4%)	
Yes, to my neighbors	111 (21.3%)	45 (22.3%)	64 (20.0%)	
Yes, to authorities/local leaders	177 (33.9%)	98 (48.5%)	79 (24.7%)	
Does not know	62 (11.9%)	13 (6.4)	49 (15.3%)	

### Physical capital

#### Food access

We explored food security using three questions from the survey, which assess quantity of food and actions taken to secure food. These were not part of a larger validated scale. The majority of respondents stated that they ate sufficiently (86.8%), but not always (57.5%), although 13.2% reported sometimes or frequently not having enough to eat, with no significant difference between migrants and non-migrants (Table 2). The most common reason for poor food security was insufficient money to purchase food. A higher wealth index was significantly associated with increased food security (*p* = 0.001), whereas migrant status was not. Although not statistically significant, almost 10% more migrants than non-migrants reported that their food supply would run out before they could acquire more. Of the 69 respondents who reported feeling food insecure, the most commonly reported coping strategies were to purchase food from the store on loan or to ration their food. While KIs never discussed food insecurity directly, they did highlight the importance of agriculture as a family’s food source. In Florida Baja, agriculture was described as “… for consumption by families.” The importance of agriculture as subsistence farming was also brought up in the FGs and described as an additional source of income when necessary.

#### Water access

A majority (84.1%) of all respondents had piped directly into their home for general and drinking use. Significantly more non-migrants had access to piped water directly in their homes than migrants (*p* = 0.046) (Table 3). Most respondents reported treating the water to make it safe to drink (69.0%), and significantly more migrants treated their water than non-migrants (*p* = 0.045). The primary method to treat water was boiling, with no major difference between groups, followed by a minority who treated with chlorine or bleach. Most KIs reported that their community had a water tank for the population and in some cases for individual households. Whether or not water was treated varied between KIs. A KI from Alegria described the availability of water: “Water … it is not potable water...it’s from a gorge, they fill a big tank for your house.” When asked where people who do not have access to this source collected water, the KI responded, “there are ravines. People go and collect their water from the ravine, but it is not potable water.” A KI from Mavila described the water service in the community saying that sometimes there is no water and when there is, the water is yellow. The KI went on to describe that the community has a tank for everyone and the health center (or municipality) treats the water with chlorine.

### Human capital

#### Health and wellbeing

A vast majority of respondents (86.1%) did seek treatment or advice from the nearest health facility when they had illnesses, with no differences between migrants and non-migrants (Table 4). Of those who sought treatment, most went to public health facilities (public hospital or health center/post). Only two people reported self-medicating, both of whom were non-migrants. Additionally non-migrants were significantly more likely to pay for their treatment than migrants (*p* = 0.004). Notably, migrants lived significantly closer to their medical treatment site than non-migrants; the majority (54.4%) of migrants reported living less than 30 min away (*p* = 0.008). Less than 10% of all respondents reported going to herbalists or traditional ‘curanderos’, with no significant difference between groups. However, the topic of traditional medicine came up in FGs, during which participants reported that coconut water was used as a treatment for both malaria and dengue.

Respondents were asked about the greatest threat to the well-being of each individual member of their household by age group. The greatest reported threat was illness (Table 5). When asked about the greatest threats to well-being, 67.6% of respondents thought illness was the greatest threat to themselves and their families followed by injury. There was a significant difference between migrants and non-migrants (*p* = 0.040) with more non-migrants reporting drugs and alcohol as threats to wellbeing.

The theme of delinquency and use of alcohol and drugs arose during a few interviews, with several KIs attributing increasing delinquency to migrants (not supported in surveys) and to the IOH construction in general. A KI from Alegria stated “Something that isn't a benefit [to the community] is the presence of migrants. Suddenly there was delinquency... Cantinas [bars] have increased, and there are people not from here that have money and drink frequently. There is prostitution [and] alcoholism.” Another KI from Santa Rosa complained, “I'm in the middle of it now; drugs and alcohol every day... [the IOH has brought us] negative things like theft, the problem of alcoholism, drugs, and prostitution.”

### Financial capital

#### Land ownership

As might be expected, migrants were more likely to own less land than non-migrants, with 75.4% of migrants owning less than one hectare, while nearly a third (28.4%) of non-migrants owned more than 20 ha (Table 6). On average, migrants owned significantly fewer hectares than non-migrants (*p* < 0.001).

One theme that came up during KI interviews, which was not explored in the survey, was the lack of land security. A KI from Florida Baja commented, "We don't feel safe because this land was sold [without guarantee] to me, so we don't feel safe in it." This was also reflected in FGs during which several community members reported that their community would be better if their property rights were recognized by the government.

#### Wealth

A small but significant difference was found between the wealth index of migrants and non-migrants, where the mean wealth score was lower for migrants than non-migrants (42.6 [95% CI: 41.5 to 43.7] vs. 47.9 [95% CI: 4.8 to 49.0], respectively; *p* < 0.0001).

### Personal capital

#### Safety

Overall, 63.0% of survey respondents reported feeling safe in their community, with no significant differences reported between migrants and non-migrants (Table 7). Interestingly, when asked about feeling more exposed to insecurity in their community in the last 5 years, a majority (58.0%) of both migrants and non-migrants reported increased insecurity, the timing of which coincides with when migration into the region began. However significantly more non-migrants than migrants reported feeling more exposed to insecurity within the past 5 years (*p* < 0.001). When asked if they had taken any additional security measures there was an almost significant difference between migrants and non-migrants (*p* = 0.051) however only 26.1% of the entire sample answered that they had employed additional security measures.

Increased feelings of insecurity have resulted in some communities coming together to form “safety committees” in which citizens comprise a neighborhood watch. A KI from Santa Rosa stated, “We have organized our own committee for citizen's security.” One KI from Florida Baja explained that in their community, committees are formed by a group of neighbors, in this specific case by 14 members, who alternate shifts to provide 24-h coverage. All are volunteers and work with the intent of preventing crime, as “sometimes there are those who don't know how to respect others’ things, and they rob them, but [now] there is a citizens’ security committee.” Only a small a minority (18%) of all residents participated in community groups, with significantly different representation of migrants and non-migrants in community organizations (14.4% vs. 23.2% respectively, *p* = 0.012).

### Social capital

#### Reasons for migration

In addition to the population of self-identified migrants, any people who were not born in the communities were also asked about their reasons for migration prior to construction of the IOH. The most commonly reported reasons for migrating to Madre de Dios were improved work opportunities and access to land for themselves or their family, followed by improved life conditions (Table 8). Work was also the most commonly reported reason for wanting to stay in the area (Table 9). Related to how well migrants feel integrated into their new home, significantly fewer migrants had their document of national identification (known locally as DNI; an identification that allows them to access state public services more easily within the documented region) assigned to their current location than non-migrants (*p* < 0.001). This was also reflected in the KI interviews, with one KI from Florida Baja stating that migrants “do not have their DNI here. They are migrants from the mountains/sierra.”

FGs, KI interviews, and survey data collectively revealed a mix of positive and negative perceptions towards migrants. The majority of non-migrant survey respondents felt that the presence of migrants had made their community worse (58.7% said migrants made it worse, 19.4% same, 21.9% better). In KI interviews and FGs, perceptions about the impact of migrants on the community were more varied. Migrants were described as having positive impacts on the community, including increased work opportunities and food availability. One KI from Alegria described the positive contributions of migrants as bringing “their culture, [and] new technology for work” to the community. However, some KI and FG participants reported increased problems with drugs and alcohol in their communities, associated with the arrival of migrants. Additionally, a third of KIs felt that migrants were not as invested in their community as local residents and therefore did not take enough care of the land or participate in the community. Another KI from Alegria summarized the mixed perceptions of migrants as, “On one hand they bring work for the population, and on the other hand, they take advantage of the population... They are businessmen that come with their money and take advantage of the humble people to buy their wood and Brazil nuts.”

#### Governance

In the survey, we asked participants which level of leadership made decisions that affected their lives. The vast majority of survey respondents thought that local and regional authorities made decisions in the community, with no differences between non-migrants and migrants (Table 10). A notable minority of respondents felt that everyone was able to make decisions through the popular vote (11.9%). Overall, there was a significant difference between what migrants and non-migrants reported regarding which level of leadership made decisions affecting their lives (*p* = 0.012); however this was because more migrants than non-migrants reported not knowing who made the decisions. Comparing local, regional and national levels of government, respondents perceived higher levels of corruption in national leaders. The majority of respondents (72.0%) believed national leaders were corrupt compared to only 42.0% of respondents who believed local leaders were corrupt. Similar trends were seen for non-migrants and migrants although significantly more non-migrants believed local leaders and regional leaders were corrupt (*p* < 0.001 and p = 0.012 respectively). Almost a third of respondents felt their opinions did not matter, while another third felt that authorities cared about their opinion. There were significant differences between migrants and non-migrants, with migrants more often feeling their opinions did not matter (*p* < 0.001). One KI from Santa Rosa described the negative aspects of her community saying, “…we are forgotten by the [national] government and we are trapped in terms of management.”

## Discussion

In this study, we defined migrants as individuals who self-identified in the survey as immigrants to the study community after the construction of the IOH—which began around 2006. Looking at when migrants moved to Madre de Dios, the largest waves of migration occurred after 2007 after the construction of the IOH had begun. This definition allowed us to examine perceptions of changes in the community associated with the construction of the IOH and the influx of migrants.

Poverty and decreased food access have been well documented in the literature [20–22]. However, in our study there was not a significant difference in access to food between migrants and non-migrants. We posit that, with the infrastructure growth due to the IOH, there was improved distribution of food in the region, decreasing issues of food access. Reduced rates of malnutrition have been reported in urban areas, suggesting that the IOH may have brought an influx of goods to Madre de Dios similar to, but on a smaller scale, than that seen in urban areas [23].

There was a significant difference in access to water, with more non-migrants having water available directly in their homes. However, direct water does not imply access to potable water. Currently there is a lack of widespread potable water in Peru, highlighting the need for government efforts to increase access to clean drinking water [24]. Access to potable water can be increased by improving household water storage and treatment which has been shown to improve water quality in low resource settings [25]. This would help decrease the threat of illness, which the majority of our participants reported as their primary threat to well-being. Improved water, sanitation, hygiene, and treatment of water with flocculant disinfectant are strongly associated with reductions in diarrheal illnesses, which are a major cause of morbidity and mortality in children under five [20, 26–28]. Interventions should focus on providing covered water vessels and educating community members on safe water treatment and storage [25].

Health access was not significantly different between groups, although migrants lived significantly closer to their point of healthcare. We posit that migrants may have moved to larger population centers due to increased economic opportunities and medical treatment sites are concentrated in these areas. We did note that many migrants had not changed their local identification card (DNI) address to their current one, and there were some misperceptions that they might not be able to access local health care. However, the results did not suggest that migrants had decreased access to health services because of this. That said, it is important to inform the communities that the public health facilities can see any patient needing assistance, not only those registered in the community. As a majority of the community feels illness is a primary threat to wellbeing, health interventions have the potential to greatly improve perceived quality of life for both migrants and non-migrants.

Various results point to the need for strengthening leadership and community capacity building in these areas. This would improve cohesion and land ownership, promote equal job opportunities, and increase the community’s involvement in their own citizen run safety committees to address real or perceived security issues. Other studies have discussed some of the challenges increased migration poses on communities, including job competition and social turmoil [29]. A study examining outward migration from rural towns in Australia posited that community self-help alone is simply not enough in these areas, and that governmental support of infrastructure, education, and employment are likely the best way to support rural growth [30]. Increasing public, private, and third party investment in growing rural communities may help improve transportation and communications, promote education and training, and create year-round meaningful employment. These macro-level changes along with the active participation of rural communities could help communities adjust to economic change and positively influence the perception of migrants as they take on a vital role in community development.

Although migrants participated significantly less in community organizations, there was minimal participation by both migrants and non-migrants in community groups. Therefore efforts to improve community integration could focus on increasing civic participation for all community members as this may provide a common meeting place and help decrease the conflict between migrants and long-term residents. Moreover, many community members, particularly migrants, lack proper/official land ownership, which likely also affects their willingness to participate in organizations. Securing land rights can often be effected through community-based land adjudication and registration, rather than expensive national programs [31, 32].

Key informants and FG participants both expressed a sense of abandonment by the national government, which may also contribute to the perception of corruption among national leaders. Survey respondents felt that local leaders were less corrupt than national leaders, suggesting the communities may be more receptive to changes from the local vs. national government. One application of this study could be to focus on future policy changes at the community level and encourage local leaders to be facilitators in order to increase community acceptance and decrease mistrust. One other study has reported perceived corruption among government leaders in Peru, although this was in relation to mining activities [5]. This study reported frustration with the limited capacity of the national and regional government to control illegal mining and foster positive feelings towards community-based conservation efforts. One reason for national government ineffectiveness proposed by a KI was the perception that lawmakers in Lima were unaware of the situation in Madre de Dios and, as a result, they pass ineffective and unenforceable laws.

There are several limitations to this study. First, this study was not designed to focus on the issue of migrants vs. long-term residents, but this topic emerged through the qualitative work. While we explored community perceptions of migrants, the voice of migrants themselves is limited, particularly in the qualitative work. The KI interviews took place with community leaders and FGs were preferentially oriented toward mothers who had lived in the community for more than five years. Due to sampling techniques key informants tended to provide introductions to other longer-term residents. As mentioned previously, the migrant and the non-migrant groups tended not to mix and as a result the voices of migrants are limited. Migrants were also more likely to be working during the day and therefore we were often unable to invite them to participate. Additionally, due to the sensitivities about illegal mining in Madre de Dios, it was sometimes difficult to find KIs willing to be interviewed and recorded, suggesting some bias in the selection of KIs. With regards to the survey, the high number of abandoned or closed houses could be a reflection of high rates of seasonal migration in these communities, which may have further contributed to the lack of migrant’s voices in this paper.

## Conclusions

Our study results suggest that efforts to improve communities affected by the IOH should not focus on migrants and non-migrants but rather the issues reported by both groups. More similarities than differences between the migrant and non-migrant groups suggest a higher level of community integration in terms of experience than is perceived. The possible applications of this research include improving access to potable water in homes as well as health services in this region, which would help ameliorate a key threat to the health and well-being reported by residents in these communities. Since there is more confidence in local leaders than the national government, changes may be best implemented by local leaders. Some of the issues to start improving include fostering community cohesion through participation in organizations, ensuring land rights, and increasing job opportunities, which could begin to break down the perceived barriers between migrant and non-migrant residents in Madre de Dios.

## Additional file

Additional file 1: Interview guide: Questions for key informants. (DOCX 83 kb)

## Abbreviations

FG: Focus group
IOH: Interoceanic highway
KI: Key informant
