# Supplementary material for: Small scale migration along the interoceanic highway in Madre de Dios, Peru: an exploration of community perceptions and dynamics due to migration
Source: BMC Int Health Hum Rights. 2018 Feb 12;18:12. doi: 10.1186/s12914-018-0152-8 (PMC5810066; doi:10.1186/s12914-018-0152-8)
Supplement: Supplementary file 1 — Interview guide: Questions for key informants. (DOCX 83 kb) [file 12914_2018_152_MOESM1_ESM.docx]

EFFECTS OF ANTHROPOGENIC HABITAT PERTURBATION ON RODENT POPULATION DYNAMICS AND RISK OF RODENT‐BORNE DISEASES

Questions for key informants

1. General information

a. When was the community established?

b. Where were the people who established the community from?

c. How many people and families live in the community?

2. People mobility

a. Are there any transient families?

i. If so, how many? How long do they stay here? What do they come for?

b. Do the children of dwellers leave the town?

i. If so, where do they go? What do they do when they leave?

c. Has there been any large movement of people coming or going from the town in the past ten years?

i. If so, could you tell us what happened?

ii. Why did people leave / come?

iii. When did that take place?

3. Economic activities

a. What are the main economic activities?

b. Have the people here always dedicated to those activities?

c. What did the people do for a living ten years ago?

4. Public services and facilities

a. Health

i. Is there a health post?

ii. Who is in charge of the health post?

iii. Do people from the town access the health post?

iv. Where do people go for health care?

b. Education

i. Are there schools? Elementary? Secondary?

ii. Is there a pre‐school? Who runs it?

c. Utilities

i. Is there electricity? If not, is there a generator?

ii. Where do most of the people in the town get drinking water? Is there a well, a reservoir? Is water treated at the community level?

iii. Is there sewage?

iv. If not, do people use latrines?

v. If there is sewage, is there any water treatment for waste water?

5. Presence of rodents

a. Does this town have any rodents, mice or rats?

b. Are they considered a problem for the community?

i. If they are, has the community done anything to curb the population of rodents?

ii. If they are, what is thought to cause the rodent problem?

c. Where do people usually see rodents: inside the households, in the surroundings of the house, in the fields, in the forest?

6. Highway

a. What are the positive and negative things the IOH has brought to the community?

b. What do you think is the most important impact (good or bad) of the IOH?

c. Have things changed in the town since the IOH was paved?

i. If so, how have they changed?

7. Organization

a. Is the community organized as an association, municipality, etc?

b. Are there any other organizations such as women’s / mother’s clubs, sports clubs, cooperatives, religious organizations, farmers associations?

c. Which are the most influential community organizations?
